# Mitochondrial genome of the mason bee, *Osmia pedicornis* (Hymenopetra: Megachilidae)

**DOI:** 10.1080/23802359.2020.1833775

**Published:** 2020-11-11

**Authors:** Hyung Joo Yoon, Jeong Sun Park, Su Yeon Jeong, Kyeong Yong Lee, Iksoo Kim

**Affiliations:** a Department of Agricultural Biology, National Academy of Agricultural Science, Rural Development Administration, Jeollabuk-do, Republic of Korea; b Department of Applied Biology, College of Agriculture & Life Sciences, Chonnam National University, Gwangju, Republic of Korea

**Keywords:** Mitochondrial genome; *Osmia pedicornis*, mason bee, Megachilidae, phylogeny

## Abstract

The mason bee, *Osmia pedicornis* Cockerell, 1919, which is importantly used as the pollinator, particularly for apples in Korea. We sequenced the mitochondrial genome (mitogenome) of *O. pedicornis* as an initial study for species identification and subsequent population genetic study. The size of the incomplete genome was 14,505 bp, excluding the *trnA*, *trnC*, and the A + T-rich region that were unable to sequence, but including partially sequenced *trnM* and *srRNA*. The genome included typical sets of protein-coding genes (PCGs), *rRNA* genes, and one non-coding region, tRNAs, excluding two unidentified tRNAs. Although positions of the two tRNAs that were not sequenced are unknown the gene arrangement of *O. pedicornis* mitogenome has the tRNA arrangement, *trnM*-*trnQ*-*trnI*, at the A + T-rich region and *ND2* junction that differed from that of previously published *O. excavate*, which has *trnA*-*trnQ*-*trnI* arrangement at the junction. Phylogenetic analyses were performed using concatenated sequences of the 13 PCGs genes and the maximum likelihood method with the inclusion of a total of 12 mitogenome sequences belonging to three families in the superfamily Apoidea. Current *O. pedicornis* was placed as the sister to the *O. bicornis*, with the highest nodal support. The Apidae and Megachilidae were placed as the sister group, with the placement of Colletidae as the basal lineage for the group with the highest nodal support.

Bees are one of the most effective pollinators accounting for 16,325 species in the world (Michener 2007). The mason bee *Osmia* Panzer, 1806 (Hymenopetra: Megachilidae) includes 339 species (Michener 2007). The mason bee differs from the honey bee in that the individuals are solitary, all females are fertile, make her own nest, and no worker bees exist (Torchio 1989; Bosch and Kemp 2001; Lee et al. 2016). *Osmia pedicornis* Cockerell, 1919 is distributed in Korea, eastern China, and Japan (Yasumatsu and Hirashima 1950) and is importantly used as the pollinator, particularly for apples (Cane 2008; Yoon et al. 2016).

In Korea, three species of *Osmia* (*O. cornifrons*, O. *pedicornis*, and *O. taurus*) are occurring, but no mitochondrial genome (mitogenome) sequences are available. Therefore, we sequenced *O. pedicornis* mitogenome as an initial study for species identification, selection of variable regions for subsequent population genetic study, and phylogenetic reconstruction of the genus and higher taxonomic groups in Apoidea to which *Osmia* is included.

In 2016, one adult of *O. pedicornis* was collected in Muneundan-ro, Nam-myeon, Jeongseon-gun, Gangwon-do, Republic of Korea (37°16′03′′ N, 128°44′29′′ E) and subsequently deposited at the Chonnam National University, Korea, under accession no. CNU12885. DNA was extracted from the hind legs of this specimen using a Wizard Genomic DNA Purification Kit (Promega, Madison, WI). Three long overlapping fragments (LFs: COI-ND4, ND5-lrRNA, and lrRNA-COI) were amplified from the genomic DNA and 28 short overlapping fragments were subsequently amplified using the LFs as templates. The primers for LFs and SFs were designed using two available *Osmia* mitogenomes (Zheng et al. 2018; Unpublished, GenBank acc. nos. KT164643, KT164653, and KT164669). A direct sequencing by Sanger’s method after PCR amplification was performed for majority of SFs, but where impossible sequencing was performed after cloning. Phylogenetic analysis was performed using 12 available mitogenomes from the clade Anthophila in the superfamily Apoidea including the one obtained in this study (Figure 1). Nucleotide sequences of 13 protein-coding genes (PCGs) were aligned and concatenated (10,993 bp excluding gaps). An optimal partitioning scheme (eight partitions) were determined using PartitionFinder 2 and the Greedy algorithm (Lanfear et al. 2012, 2014, 2016). Maximum likelihood (ML) analysis was performed using RAxML-HPC2 on XSEDE version 8.0.24 (Stamatakis 2014), implemented on the CIPRES Portal version 3.1 (Miller et al. 2010).

**Figure 1.**
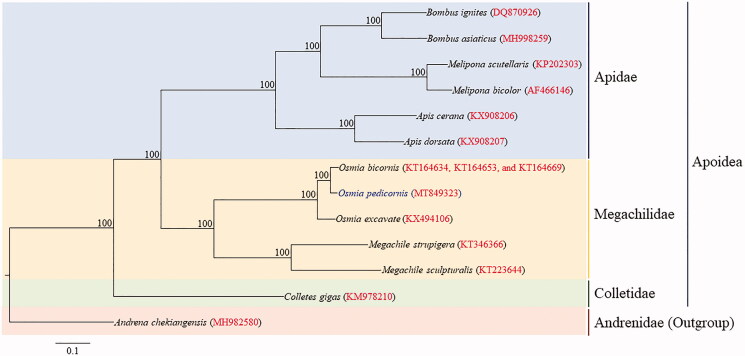
Maximum likelihood (ML) method-based phylogenetic tree for the superfamily Apoidea using concatenated sequences of 13 protein-coding genes. The numbers at each node specify the bootstrap percentages of 1000 pseudoreplicates. The scale bar indicates the number of substitutions per site. *Andrena chekiangensis* belonging to the family Andrenidae in the superfamily Apoidea (MH982580, He et al. 2019) was utilized as an outgroup. The GenBank accession numbers are as follows: *Bombus asiaticus*, MH998259 (Zhao et al. 2019); *B. ignitus*, DQ870926 (Cha et al. 2007); *Apis cerana*, KX908206 (Wang et al. 2018); *A. dorsata*, KX908207 (Wang et al. 2018); *Melipona bicolor*, AF466146 (Silvestre et al. 2008); *M. scutellans*, KP202303 (Pereira et al. 2016); *Osmia pedicornis*, MT849323 (This study); *O. excavata*, KX494106 (Zheng et al. 2018); *O. bicornis*, KT164634, KT164653, and KT164669 (Unpublished); *Megachile sculpturalis*, KT223644 (Zhang et al. 2017); *M. strupigera*, KT346366 (Huang, Su, He, et al. 2016); and *Colletes gigas*, KM978210 (Huang, Su, Qu, et al. 2016).

Although a substantial attempt to sequence whole mitogenome of the *O. pedicornis* was made we eventually were unable to sequence a serial genes located around the A + T-rich region, such as *trnA*, *trnC*, *trnM* (partially), and *srRNA* (partially) including the A + T-rich region. Probably this may happen due to the unexpectedly long A + T-rich region, which possibly contains the repeat sequences and higher A/T nucleotide, along with nonspecific amplification. In fact, the *O. excavata* A + T-rich region expands to 1472 bp, but is still incomplete and contains several repeat sequences, ranging in size from 9 to 38 bp (Zheng et al. 2018).

The size of the *O. excavata* mitogenome was 14,505 bp, excluding the *trnA*, *trnC*, and the A + T-rich region that were unable to sequence, but including partially sequenced *trnM* and *srRNA*. The size and A/T content of the *O. pedicornis* PCGs was 3684 codons (excluding termination codons) and 83.9%, respectively, and are similar to that of *O. excavata* (3682 codons and 82.4%, respectively). The size and A/T content of *O. pedicornis lrRNA* was 1328 bp and 86.2% and also is similar to those of *O. excavata* (1320 bp and 85.8%, respectively).

The gene arrangement of *O. excavata* mitogenome, which lacks for the information for two tRNA positions (*trnA* and *trnC*) differed from that of *O. excavata* mitogenome (Zheng et al. 2018) in that the five tRNA region located at the A + T-rich region and *ND2* junction has an *trnM*-*trnQ*-*trnI* arrangement, instead of the *trnA*-*trnQ*-*trnI* arrangement in *O. excavate* (Zheng et al. 2018).

Phylogenetic analysis using nucleotide sequences of 13 PCGs with the representative mitogenome sequences of Apoidea showed that each Apidae and Megachilidae, which were represented by multiple sequences formed monophyletic groups with the highest nodal supports (Figure 1). Among three families represented for Apoidea the Apidae and Megachilidae were placed as the sister group, leaving Colletidae as the basal lineage for the group with the highest nodal support. Within Megachilidae, to which *Osmia* is included three species of *Osmia* formed a monophyletic group with the highest nodal support, forming *O. pedicornis* and *O. bicornis* the sister group. This *Osmia* group was placed as the sister to two *Megachile* species. A recent whole genome sequence-based phylogenetic analysis including several families of Apoidea has also shown the sister relationship between Apidae and Megachilidae with the placement of Colletidae as the basal lineage for the group (Zhou et al. 2020), presenting an identical phylogenetic relationship to current results (Figure 1).

## Data Availability

The genome sequence data that support the findings of this study are openly available in GenBank of NCBI at https://www.ncbi.nlm.nih.gov/nuccore/MT849323.1
